# Air Pollution Impact on Pregnancy and Early Childhood Development (APiPED) in India: Protocol for a Cohort Study

**DOI:** 10.2196/72683

**Published:** 2025-11-11

**Authors:** Harshal Ramesh Salve, Sagnik Dey, Yashika Arora, Surbhi Kapoor, Santu Ghosh, Rakesh Kumar, Sheffali Gulati, Rajesh Sagar, Sudip Kumar Datta, Anand Krishnan

**Affiliations:** 1 Centre for Community Medicine All India Institute of Medical Sciences New Delhi, Delhi India; 2 Collaborative for Air Pollution and Health Effects Research (CAPHER)-India New Delhi, Delhi India; 3 Centre for Atmospheric Sciences Indian Institute of Technology Delhi New Delhi, Delhi India; 4 Centre of Excellence for Research on Clean Air Indian Institute of Technology Delhi New Delhi, Delhi India; 5 St John's Research Institute Bengaluru, Karnataka India; 6 Child Neurology Division All India Institute of Medical Sciences New Delhi, Delhi India; 7 Department of Psychiatry All India Institute of Medical Sciences New Delhi, Delhi India; 8 Department of Laboratory Medicine All India Institute of Medical Sciences New Delhi, Delhi India

**Keywords:** air pollution, pregnancy, longitudinal study, particulate matter, exposure assessment, child development, neurodevelopment cohort studies, India

## Abstract

**Background:**

The impact of air pollution on early childhood development in low-pollution settings is well known. However, comprehensive evidence from high-pollution environments such as the Delhi National Capital Region in India remains limited.

**Objective:**

This maternal-child cohort study aims to investigate the impact of air pollution on pregnancy outcomes and early childhood development, providing critical insights to inform targeted interventions and public health policy.

**Methods:**

This longitudinal maternal-child cohort study will enroll 2500 pregnant women from rural and urban Delhi National Capital Region and follow their children up to 2 years of age. Maternal data on pregnancy complications, delivery mode, parity, and gestational weight gain will be collected from clinical records and structured questionnaires, while newborn outcomes (gestational age, birth weight, anthropometry, and congenital anomalies) will be assessed from birth records and clinical examination. Early childhood development will be evaluated through standardized anthropometry and the Developmental Assessment Scale for Indian Infants. For a 10% subsample, trimester-specific and postnatal indoor and outdoor air pollution exposure (particulate matter with a diameter of less than 2.5 micrometers and particulate matter with a diameter of less than 10 micrometers) will be monitored using portable air quality monitors, along with household surveys and time-activity diaries. Maternal and infant blood samples will be analyzed for inflammatory, oxidative stress, and cardiovascular biomarkers. Exposure estimates will be integrated into personal exposure models, and associations with health outcomes will be examined using multivariable regression and longitudinal mixed-effects models.

**Results:**

As of July 2025, 45% of the planned sample size have been recruited, with baseline data collection completed, and 10% undergoing exposure assessment and sample collection.

**Conclusions:**

This study will provide a comprehensive evaluation of the effects of air pollution on maternal health, pregnancy outcomes, and early childhood development in urban and rural settings in India. This will generate context-specific evidence to support maternal and child health policies, air pollution mitigation strategies, and personal protection measures for pregnant women and infants in polluted environments.

**International Registered Report Identifier (IRRID):**

DERR1-10.2196/72683

## Introduction

Environmental risk factors, particularly air pollution, contribute to over 2 million deaths annually in India [1]. The source share of household air pollution in these attributable deaths is equivalent to the ambient air pollution. According to the Global Burden of Disease Study 2017, approximately 76.8% of the Indian population lives in regions exceeding the Indian National Ambient Air Quality Standard (NAAQS) for PM_2.5_ (40 µg/m^3^), leading to an estimated 1.24 million deaths (95% UI 1.09–1.39 million) attributable to air pollution in 2017 [2]. Older adults, children and pregnant women are particularly vulnerable to adverse effects from air pollution. For women and children, indoor activities and household cooking with solid fuels increase exposure to fine particulate matter and carbon monoxide [3]. Pregnant mothers carrying developing embryos and fetuses, along with newborns and infants, present high and unique susceptibilities to these health risks posed by airborne pollutants. In northern India states such as Delhi, Uttar Pradesh and Haryana, annual PM_2.5_ exposures exceeded 125 µg/m^3^ (more than three times the standard) [2]. While the health burden of high exposures in those settings is substantial, evidence on specific long-term outcomes such as child growth or lingering effects from prenatal exposure is still emerging. Several studies suggest that air pollution is associated with low birth weight [4,5], but whether that association persists as children grow remains to be fully elucidated.

In India, the impact of air pollutants on growth and early childhood development is largely unknown. Evidence regarding the impact of the health status of the mother during pregnancy and newborn outcomes is available [6,7]. These studies are restricted to socioeconomic, biological, clinical, and behavioral characteristics in mothers and their effects on birth outcomes. Existing research studies do not adequately address the environmental factors contributing to adverse birth outcomes. The evidence on the association between birth outcomes and air pollution exists in the form of studies correlating cooking fuel type as a proxy of household air pollution with low birth weight outcomes [8,9]. In a few cohort-based studies, the research documents biomass smoke with secondhand tobacco smoke as a determinant for not only low birth weight but also infant mortality and child respiratory morbidity [10]. There is limited quantitative evidence in India linking rural-urban PM_2.5_ exposure to birth weight. Nutritional outcomes and developmental milestones play an important role in child survival and healthy adulthood. A prospective cohort approach is an appropriate way to assess the temporality of association between air pollutant exposure and child health outcomes. Experimental and in vitro studies suggested that endocrine disruption, oxidative stress, inflammatory response, and DNA damage may be contributing to adverse pregnancy and birth outcomes associated with maternal air pollution exposure. Hence, exploring possible metabolic biomarkers using a prospective design and repeated measures becomes important to understand the causal mechanism.

The study aims to understand how long-term exposure to air pollution during pregnancy and early childhood can impact pregnancy and neonatal outcomes, and early childhood development, respectively. The other objectives of the study are to examine the relationship between long-term indoor and outdoor air pollution exposure during pregnancy and pregnancy outcomes among rural and urban populations in the National Capital Region (NCR), India. The study will also analyze the role of blood biomarkers as mediators in the pathway between air pollution exposure and its effects on pregnancy outcomes and early childhood development.

The conceptual framework for this study is structured around 4 core domains. The environmental exposure domain encompasses exposure to PM levels in both indoor and outdoor environments. The biological mediation domain addresses the underlying biological pathways, including oxidative stress and inflammatory responses, and focuses on biomarkers such as interleukin-6 (IL-6), tumor necrosis factor (TNF) alpha, and cardiometabolic indicators such as C-reactive protein. The developmental and health outcomes domain captures maternal and child neurodevelopmental trajectories, while the sociodemographic and environmental modifiers domain considers effect modifiers and potential confounders, including socioeconomic status, diet, breastfeeding practices, parental education, housing conditions, cooking fuel use, access to health care, and prenatal care. The theoretical underpinning of this framework is grounded in the Developmental Origins of Health and Disease [11] theory, which postulates that environmental exposures during critical windows of development, such as gestation and early infancy, can shape long-term health and developmental outcomes. This framework integrates quantitative measures of environmental exposure, biomarker-based mediators, and developmental outcomes, while also explicitly capturing the temporal sequence of exposure, mediation, and outcome, thereby enabling a mechanistic understanding of how early-life environmental exposures influence neurodevelopmental health.

## Methods

### Overview

The proposed study is a prospective, population-based mother-child cohort that will be assembled in both rural and urban settings. The study population will consist of mother-child pairs from the general population. Part of the sample will come from a rural area, and another part will come from an urban site. The study will build on the existing cohort in rural and urban demographic surveillance sites [12]. The rural site is located in the Ballabgarh Block of Faridabad district, and the urban site is located in Dr Ambedkar Nagar of the South-East district of Delhi NCR, India. Both rural and urban study sites have been under demographic surveillance for the last 3 decades by the Centre for Community Medicine of All India Institute of Medical Sciences (AIIMS), New Delhi. Under health demographic surveillance, each birth, death, and other health outcomes of the mother and newborn child are recorded and stored in a computerized health and management information system. This facility will robustly help recruit cohorts. Continued presence of health services from the institutions will ensure maximum community support during the study.

### Participants

Study participants will be PW and children (birth to 24 months) born to the recruited PW residing in the study sites. The study population will mostly belong to the middle and low socioeconomic classes living in the stable cohort, both at rural and urban sites. The study has adopted a multipronged strategy to ensure retention in the air pollution impact on pregnancy and early childhood development cohort. As part of the protocol, the inclusion criterion requires participants to have a minimum of 2 years of residence at the demographic sites. Furthermore, retention is strengthened through community engagement mechanisms that leverage existing systems in demographic surveillance sites, that is, through Accredited Social Health Activists and Anganwadi workers. The front-line workers maintain regular contact with the participants through scheduled home visits during pregnancy and postnatal periods, aligning with national health program protocols. The risk of dropouts is further reduced as the study uses flexible scheduling of participant home visits by field workers. Moreover, the visits are strategically aligned with routine community-based events such as Village Health Nutritional Days and immunization schedules at health care centers in both rural and urban sites.

### Data Collection

During the initial visit to the households of eligible and consenting PW, detailed residential information (including contact details and geographic coordinates), as well as information about the participant and her household members, will be collected. This baseline information will gather data on the woman’s age, education, occupation, lifestyle factors, medical history, and relevant family history. In addition, demographic, socioeconomic, and environmental factors about the household will be collected. For a comprehensive analysis, additional baseline information about PW will be gathered using secondary data available in records. All interview data, secondary information, and monitoring records will be collected using the Kobo Collect App (Kobo Organization) and stored in the KoboToolbox Cloud (Kobo Organization; [13]).

PW will be contacted every 3 months (each trimester) to collect follow-up information on events during pregnancy or hospitalization. One ultrasonography record (possibly in the second trimester) will be retrieved from the recruited PW for assessing fetal well-being. Delivery outcomes will be assessed by visiting PW immediately after delivery. Based on past hospital records, the institutional delivery rate in the study area is nearly 100%. The validity of information on birth outcomes will be checked from the hospital records and during home visits. A detailed list of study forms, along with a brief outline of the content of each questionnaire, is provided in Multimedia Appendix 1. The study plans to undertake exposure monitoring and biomarker analyses in 10% of cohort participants owing to financial constraints and feasibility considerations. In rural sites, participants will be selected using proportionate probability sampling at the village level, while in urban sites, systematic random sampling will be applied to capture heterogeneity. This subsampling strategy ensures representativeness, is resource-efficient, and enables scientifically valid exposure-response assessments. Nested subcohort and case-cohort designs are well established in epidemiological research and have demonstrated that, when sampling is systematic and appropriately stratified, robust estimates of exposure-outcome associations comparable to those from full cohorts can be obtained [14,15].

Personalized exposure assessments to air pollution will be conducted in 10% of the recruited participants, 3 times during pregnancy (once in each trimester) for PW and every 6 months for 2 years for their newborns. Recruited newborns will be visited every 6 months from birth until the age of 24 months for the assessment of childhood outcomes. In addition, pediatricians on the project team will assess various nutritional and physiological outcomes among the recruited children. Blood samples (from 10% of recruited PW and newborns) will be collected at baseline and in the third trimester for PW, and after 6 months and 24 months for the newborns, for the assessment of biomarkers. In total, for both PW and the newborns, 900 blood samples will be taken to have repeated measures of biomarkers. Enzyme-linked immunosorbent assay kits will be used to detect and quantify biomarkers in blood samples, using the in-house facilities at the Department of Laboratory Medicine, AIIMS, New Delhi.

**Figure 1:**
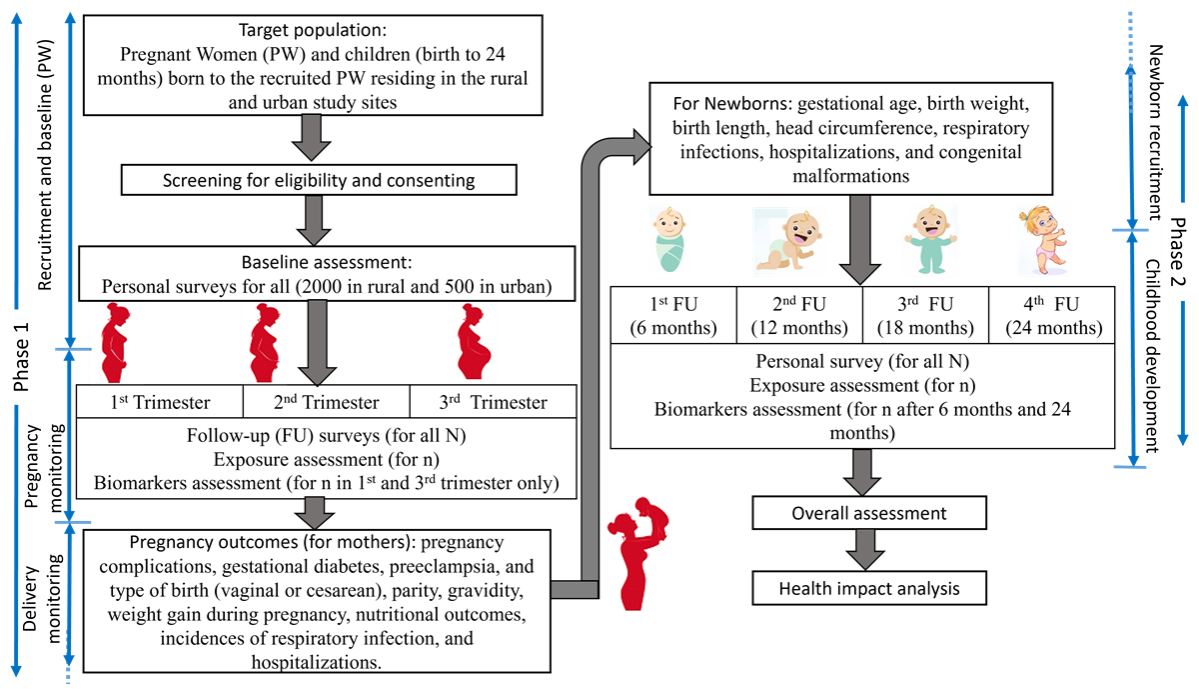
Study flow outlining the longitudinal phases of the air pollution impact on pregnancy and early childhood development (APiPED) dynamic cohort. Phase 1 includes the recruitment of pregnant women (PW) from rural (N=2000) and urban (N=500) sites, baseline assessments, trimester-specific follow-ups, exposure and biomarker assessments, and monitoring of pregnancy outcomes. Phase 2 follows newborns through 4 follow-ups (at 6, 12, 18, and 24 months), assessing growth, health outcomes, exposure, and biomarkers. The study concludes with an overall health impact assessment and analysis.

The study design consists of 2 phases. Phase 1 will focus on recruiting PW from rural (n=2000) and urban (n=500) sites, conducting baseline assessments, and performing follow-ups during each trimester to monitor exposure, biomarkers, and pregnancy outcomes. Phase 2 will involve follow-up assessments of children at 6, 12, 18, and 24 months to evaluate growth, health outcomes, exposure, and biomarkers. The study will conclude with an overall assessment and health impact analysis. The study flow, consisting of longitudinal phases, is presented in Figure 1.

### Exposure Assessment

A hybrid exposure model will be developed combining indoor, outdoor, and ambient PM_2.5_ measurements along with satellite-based PM_2.5_ fused with a land use regression (LUR) model to estimate the pregnancy period as well as life course exposure of the children. We will use indoor and outdoor air quality monitors to measure PM_2.5_, temperature, and relative humidity for 10% of PW, assessing their indoor and outdoor air pollution exposure over 24 hours during each trimester. The same monitors will also track the air pollution exposure of newborns for 24 hours across all seasons (every 6 months over a period of 2 years). Personal exposure levels will be reconstructed by combining the participants’ time-activity profiles with both outdoor (satellite-derived and ground-based) and indoor measurements.

The levels of PM of various sizes and other pollutants at each study site will be obtained from multiple sources. Using a high-resolution (1-km, 24-hour average) PM_2.5_ database from the satellite-based application for air quality monitoring and management at a national scale (SAANS) project [16]—designed for air quality management, epidemiological research, and public awareness—the spatial patterns of PM_2.5_ for the study sites in the Delhi-NCR area will be evaluated.

Throughout the study, we will collect hourly mean values of air PM_10_ and PM_2.5_ for 24-hour periods from the Central Pollution Control Board website, where open-access data from fixed-site automatic monitors in the Continuous Ambient Air Quality Monitoring Stations network are available [17-19]. In addition, we will gather 5 years (2019-2023) of historical data on daily and annual average PM recorded by Continuous Ambient Air Quality Monitoring Stations and manual gravimetric samplers as part of the National Air Quality Monitoring Program. We will further extract parameters of LUR, such as proximity to line and point source emissions, traffic density, land use pattern, vegetation index, and population density, within a 1x1 km^2^ buffer of the cohort using the Arc Geographic Information System following the methodology applied in previous studies [20-22].

**Figure 2:**
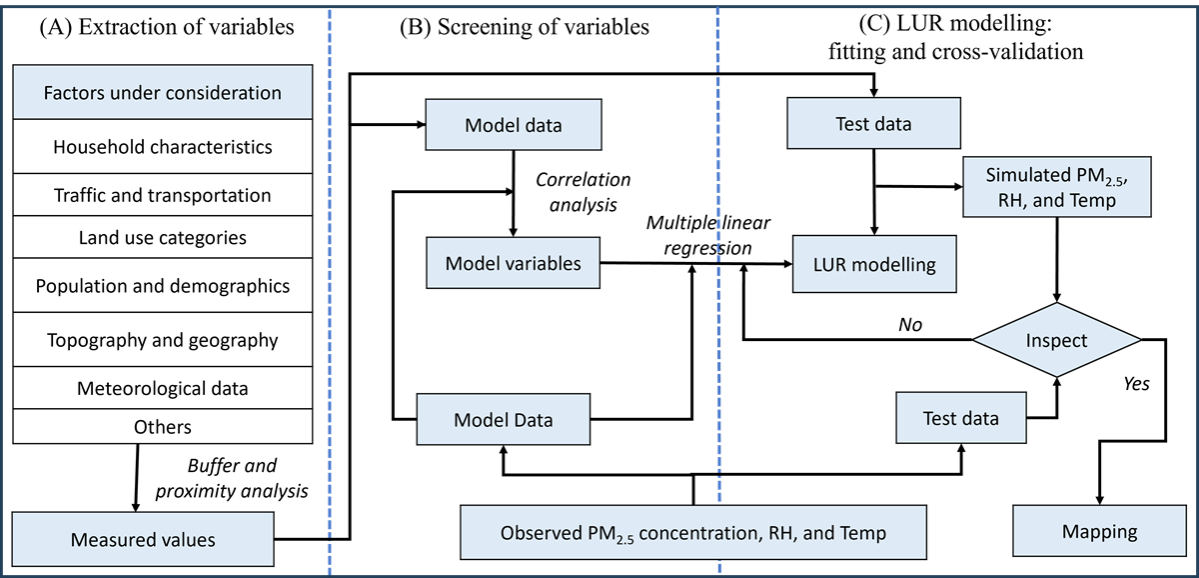
Implementation scheme for exposure modeling using the land use regression (LUR) method in the air pollution impact on pregnancy and early childhood development (APiPED) cohort study. (A) Extraction of variables from various sources. (B) Screening of selected variables for relevance. (C) LUR model development and validation process, including fitting and cross-validation techniques. PM: particulate matter; RH: relative humidity; Temp: temperature.

In developing our LUR model, we will incorporate a variety of geographic, demographic, and emission source–specific predictor variables sourced from health records, personal surveys, open-access databases, local authorities, and field verification. This will include land use types (residential, industrial, etc), road network characteristics, population density, elevation, and potential emission sources. Satellite-derived aerosol optical depth and meteorological reanalysis data will also serve as predictors. We will use a supervised forward addition linear regression technique for model development and conduct diagnostic tests to ensure the integrity of the regression. The model’s performance will be evaluated using a 10-fold cross-validation approach [23,24]. The implementation scheme is presented in Figure 2.

Finally, we will apply this hybrid exposure modeling approach to estimate long-term personal exposure to PM during pregnancy as well as across the life course for all study participants, using geocoded locations and other relevant predictors within the model.

### Outcomes

The primary exposure or independent variables examined in this study will be the mean concentrations of PM_2.5_ and PM_10_, temperature, and relative humidity measured longitudinally using personal indoor and outdoor monitors and centralized sources, as described in the “Exposure Assessment” section.

The primary study outcomes for assessing the impact of air pollution are tabulated in Textbox 1. The study will collect the following pregnancy outcomes for mothers: pregnancy complications, gestational diabetes, preeclampsia, type of birth (vaginal or cesarean), parity, gravidity, and weight gain during pregnancy. For newborns, the following outcomes will be noted: gestational age, birth weight, birth length, head circumference, and congenital malformations. Assessment of developmental milestones will be conducted using the Development Assessment Scales for Indian Infants [25]. This is an Indian adaptation of the Bayley Scales and measures the developmental quotient for the age group 1-30 months. Development Assessment Scales for Indian Infants is the most widely used scale in India, culturally appropriate, and standardized for Indian infants, whereas the Bayley Scales are better suited for the US population. It is an effective, comprehensive, cost-efficient, and practical tool. Three key developmental domains that will be monitored are cognition, language, and motor development. Other developmental outcomes include peak height and weight growth velocities.

Biomarker assessment will be conducted in 10% of the study sample for profiling of inflammation, cardiovascular disease, and oxidative stress [26]. Blood samples from PW (at baseline, 6 months, and 2 years post-pregnancy) and from children (at 6 months and 2 years of age) will be collected. In this study, the role of various inflammatory biomarkers potentially associated with air pollution exposure will be assessed. These include proinflammatory cytokines such as IL-1, IL6, IL-8, TNF-α, anti-inflammatory cytokines (IL-10, transforming growth factor β), anti-infectious (interferon γ, TNF-α), B-cell–activating cytokines (IL-3, IL-4, IL-5, IL-6, IL-21), and T-cells–activating cytokines (IL-2, IL-4, IL-10, IL-13, IL-15) [27]. In addition, the study will evaluate cytokines indicative of cardiovascular risk associated with air pollution, including endothelial adhesion molecules (soluble intercellular adhesion molecule-1 and soluble vascular cell adhesion molecule-1), plasminogen activator inhibitor-1, C-reactive protein, and myeloperoxidase [28].

**Outcomes for assessing the impact of air pollution in the air pollution impact on pregnancy and early childhood development (APiPED) cohort. The primary outcomes include pregnancy, neonatal, and early childhood development outcomes.**

**Pregnancy outcomes:**

- Pregnancy complications
- Gestational diabetes
- Preeclampsia
- Type of birth (vaginal or cesarean)
- Parity
- Gravidity
- Weight gain during pregnancy

**Neonatal outcomes:**

- Gestational age
- Birth weight
- Birth length
- Head circumference
- Congenital malformations

**Early childhood development outcomes**:

- Motor domain (using Developmental Assessment Scale for Indian Infants [DASII]):
  Neck control
  Body control
  Locomotion-1
  Locomotion-2
  Manipulation
- Mental domain (using DASII):
  Cognizance (visual)
  Cognizance (auditory)
  Reaching and manipulation
  Memory
  Social interaction and imitative behavior
  Language 1 (vocalization, speech, and communication
  Language 2 (vocabulary and comprehension)
  Understanding the relationship
  Differentiation by use, shape, and movement
  Mental dexterity
- Peak growth velocities for height
- Peak growth velocities for weight

### Study Sample

The rural site consists of a population of 110,000, while the urban site comprises a population of 50,000. This will yield approximately 2000 PW at the rural site and 500 at the urban sites in a year. All pregnant mothers at both sites will be recruited (total 2500 PW). Pregnant women will be followed for birth outcomes such as low birth weight and premature births. The selection of the cohort of PW will be based on continuous monitoring of pregnancies in the rural and urban sites throughout the year using the existing health and demographic surveillance data at study sites during 2024-2025 and periodic screenings. The sampling time frame will be reinforced by conducting household visits to ensure accurate data collection. Newborn children, accounting for a 10% attrition rate of recruited pregnancies, will be followed for 2 years every 6 months to assess nutritional outcomes, developmental milestones, and psychological assessment. Information on acute respiratory morbidities, biomarker assessment, social determinants, and macroenvironment details will be collected at baseline and follow-up. Only women residing in the study area for at least 1 year and willing to remain in the study area for the study period will be included.

### Implementation Strategy

All data collected in the project will adhere to confidentiality and anonymization protocols to protect participant privacy. Personally identifiable information, including names, addresses, and contact details, will be collected at enrollment and stored in a secure, access-controlled database. Each participant will be assigned a unique study identification number, which will be used across all forms, questionnaires, blood samples, and electronic records. This ensures that no personally identifiable information is linked to clinical, exposure, or biomarker data during analysis. Electronic data will be stored on encrypted servers with role-based access control. For dissemination, only deidentified and aggregated data will be reported. For blood samples, the Unique Health Identification system by AIIMS will be used. Samples will be labeled only with the study identification, Unique Health Identification, and collection date. All laboratory personnel analyzing samples will be blinded to participants’ identities and exposure status.

For exposure assessment, monitors will be calibrated regularly and rotated to assess drift. In the event of personal monitor malfunction or incomplete capture (<80% valid readings/day), the affected measurements will be flagged, and repeat sampling will be undertaken where feasible. If not feasible, exposures will be imputed using the nearest Central Pollution Control Board station data adjusted for microenvironment or modeled values from the LUR–satellite hybrid framework. In addition, fixed outdoor monitors are deployed at health care centers in both rural and urban sites to provide continuous background reference data. For biomarker analysis, calibrated and quality-checked instruments and high-quality reagents will be used. Research staff will undergo thorough training, and questionnaire-based data collection will rely on standardized tools (KoboToolbox and Google Sheets), with any newly developed tools validated before deployment.

The project’s strong urban-rural cohort infrastructure, supported by a multidisciplinary research team comprising experts in public health, social work, pediatric neurology, psychiatry, laboratory medicine, and exposure assessment, will significantly strengthen its ability to collect high-quality data and deliver meaningful outcomes. Standardized protocols and training by subject experts will be provided to the field team to accurately measure environmental exposure and health outcomes, reducing errors and minimizing biases.

For childhood development outcomes, pediatricians in the project team will be engaged to assess various nutritional and physiological outcomes among the recruited children. Developmental assessment at each visit will be done by the trained field worker using valid methods. Training will be provided by a psychiatrist in the investigator team from AIIMS, New Delhi.

### Statistical Analysis

Statistical analysis will be carried out in standard statistical software, such as R (R team and the R Foundation), Stata (StataCorp), and SPSS (IBM Corp). The association between air pollution exposure and pregnancy outcomes will be explored using a linear or logistic regression model, depending on the nature of the outcome measure. On the other hand, anticipating a negatively skewed distribution of the score developmental tools, either Beta Binomial regression [29] or logistic regression (after dichotomizing the scores; [30]) will be applied to estimate the impact of air pollution on child development. The association between air pollution and biomarkers will be examined using a linear or log-linear mixed-effects model due to their longitudinal nature. The rural and urban subsamples will be analyzed separately and then meta-analyzed. Continuous and quartile-based analyses of exposure assessment variables (PM_2.5_ and PM_10_) will be conducted to explore their effects on health outcomes during pregnancy and early childhood.

### Confounders and Effect Modifiers

The study will collect the following sociodemographic characteristics: age of parents, family composition, parental education, financial situation, occupation, religion, type and size of home, floor, and exposure to indoor pollution. The study will also collect the following lifestyle factors: breastfeeding, second-hand smoke exposure, alcohol consumption during pregnancy, time spent outdoors, diet, physical activity, heating and cooking modes at home, pets, and nursery attendance. The following physical environmental factors will be collected: pesticides, per- and polyfluoroalkyl substances, phthalates, phenols, stubble burning, and meteorological factors. Finally, data on stressful events during pregnancy and early life of the child, residential and workplace history, information about infectious diseases, and medications will be collected.

Directed acyclic graphs will be used to visualize conceptual models and determine confounders that need adjustment in regression models. All regression models will be adjusted for relevant confounders, including sociodemographic variables (maternal age, education, and income), reproductive history (parity and gravidity), environmental factors (indoor fuel use and season), and lifestyle factors (tobacco smoke exposure, diet, and physical activity). For developmental outcomes, postnatal exposures, such as breastfeeding, recent infections, and nutritional status, will also be included in the adjustment models.

All effect estimates will be reported with 95% CIs. Statistical significance will be defined as a 2-sided *P*<.05. Sensitivity analyses will be conducted to test the robustness of findings, including copollutants, temperature, and stratification by urban or rural setting, sex of the child, or socioeconomic status.

### Ethical Considerations

Ethical approval for this study was obtained from the Institute of Ethics Committee of the AIIMS, New Delhi (ref no AIIMSA00452/12.01.2024). All biosafety procedures will be followed in handling and storing the biological samples. The results of blood testing will be communicated to the study participants. Informed consent will be obtained from PW for data collection and dissemination through scientific publications, covering both their participation and that of their child. Participant information will be anonymized to ensure confidentiality during data analysis and publication.

## Results

The air pollution impact on pregnancy and early childhood development (APiPED) project received approval from the Indian Council of Medical Research, New Delhi, in November 2023. Institutional ethical approval was obtained in January 2024, followed by operative approval in February 2024. The recruitment of project personnel began in April 2024, and the project team includes research scientists and project technical support personnel working under the supervision of project investigators and coinvestigators.

For personal exposure assessment, data collection tools, indoor and outdoor air monitors, and equipment have been procured. In October-November 2024, training sessions for field staff were conducted, along with equipment procurement and monitor calibration. Since project initiation, refresher trainings have been conducted every 4 months for project technical support personnel to maintain consistency and minimize variability in data collection. Participant recruitment began in December 2024. As of July 2025, 45% of the participants have been recruited, and baseline data collection has been completed. In addition, personal exposure assessment and sample collection have been conducted for 10% of the recruited participants.

## Discussion

### Principal Findings

The proposed study is a prospective, population-based cohort that combines data from rural and urban settings to examine the association between air pollution exposure and outcomes, considering temporal relationships. For pregnant mothers, outcomes include pregnancy results (abortion, stillbirth, premature birth, or normal birth), nutritional status, and incidences of respiratory infections and hospitalizations. For children, outcomes include mortality, birth weight, respiratory infections, hospitalizations, developmental milestones, early childhood development, and nutritional indicators such as underweight and stunting. This study will identify air pollution exposure models and associated blood biomarkers for pregnancy and child health in rural and urban sites of Delhi NCR. While the impact of air pollution on early childhood development is well-documented in low-pollution settings, evidence from high-pollution areas such as Delhi NCR will provide critical insights into its developmental and generational effects. Such findings will support policymakers and industry partners in implementing targeted interventions.

### Strengths

One of the key strengths of this study is the use of personalized and portable air pollution exposure assessments. The study will use both indoor and outdoor monitoring of air pollution, which ensures a more accurate evaluation of the exposure levels faced by participants [31]. Integrating satellite-derived, ground-based, and personal exposure measurements to construct 24-hour exposure profiles will enhance the precision of the exposure models [32]. This personalized exposure assessment will be crucial in linking air pollution levels to the health effects observed in the cohort. The study features a comprehensive framework that evaluates a wide range of pregnancy and early childhood development outcomes, alongside exploratory biomarker assessments. Furthermore, integration with ongoing health and demographic surveillance at the study sites will enable long-term monitoring of participants, ensuring comprehensive follow-up into later childhood development.

### Limitations

Despite the study’s strengths, there are a few limitations. First, the impact of air pollution on pregnancy and early childhood developmental outcomes is primarily considered through PM_2.5_ and PM_10_ effects. Due to limited resource settings, other air pollutants are not measured for personalized monitoring. In addition, while the study will use personal monitors to measure air pollution exposure, these monitors will collect data for a limited duration. Given the variable nature of air pollution, this may not fully capture long-term exposure patterns. Furthermore, the study is subject to the risk of confounding factors such as socioeconomic status, nutrition, and pre-existing health conditions that may influence the observed outcomes. All analyses will account for various confounding variables included in the study.

One of the primary challenges for the proposed study is ensuring consistent and high-quality data collection across both rural and urban sites. Recruitment and retention of study participants can also be challenging, as people in low-resource settings may have limited availability or interest in research participation. Furthermore, variability in air pollution exposure, both spatially and temporally, can make it difficult to assess the precise effects of specific pollutants on health outcomes. This requires careful consideration of the exposure assessment methodology and the need for real-time data to improve the accuracy of exposure models. Another challenge is the collection of comprehensive data over long periods, particularly regarding early childhood development. Regular follow-up visits are planned in the study to track developmental milestones and ensure the accurate assessment of outcomes such as stunting, underweight, and cognitive development. The proposed study will provide standardized protocols and training by subject-matter experts to the field team to accurately measure environmental exposure and health outcomes, thereby reducing errors and minimizing biases.

### Practical Implications

The findings from this study will have significant practical applications. By providing robust evidence on the effects of air pollution on maternal and child health, the study will be instrumental for policymakers working toward meeting Sustainable Development Goal 3, which promotes good health and well-being. The study aligns with the priorities of the National Health Mission in India, particularly in addressing environmental factors that contribute to noncommunicable diseases and ensuring the health of vulnerable populations. Moreover, the biomarkers developed through this study will provide a tool for future research on the environmental impacts of air pollution, offering an opportunity to refine epidemiological models and improve public health interventions. The project tools, software, and frameworks will be thoroughly documented and shared as open-source resources for other researchers.

### Conclusion

The proposed study aims to address a critical gap by providing direct evidence on the impact of air pollution on childhood development in India. A personal exposure model will be developed by integrating participants’ time-activity profiles with both outdoor and indoor measurements. In addition, it will offer a framework for personal exposure modeling at the community level in both rural and urban settings. The study will also investigate the potential of biomarkers for predicting the effects of air pollution during pregnancy and early childhood. By investigating both immediate and long-term effects, this study will provide valuable data to inform policy decisions, promote public health awareness, and support the development of effective interventions. The results will not only enhance scientific understanding but also provide actionable insights to mitigate the adverse effects of air pollution on vulnerable populations.

## Appendix

Multimedia Appendix 1 Study forms and questionnaires.

## Abbreviations

AIIMS: All India Institute of Medical Sciences
APiPED: air pollution impact on pregnancy and early childhood development
IL: interleukin
LUR: land use regression
NCR: National Capital Region
PM: particulate matter
PW: pregnant women
SAANS: satellite-based application for air quality monitoring and management at a national scale
TNF: tumor necrosis factor
