# Supplementary material for: Air Pollution Impact on Pregnancy and Early Childhood Development (APiPED) in India: Protocol for a Cohort Study
Source: JMIR Res Protoc. 2025 Nov 11;14:e72683. doi: 10.2196/72683 (PMC12648132; doi:10.2196/72683)

**A. List of Forms**

1. Consent Form of pregnant women
2. Baseline form
3. Follow Up Form of pregnant women
4. Exposure Assessment form
5. New Born Child Form

**B. Form Details:**


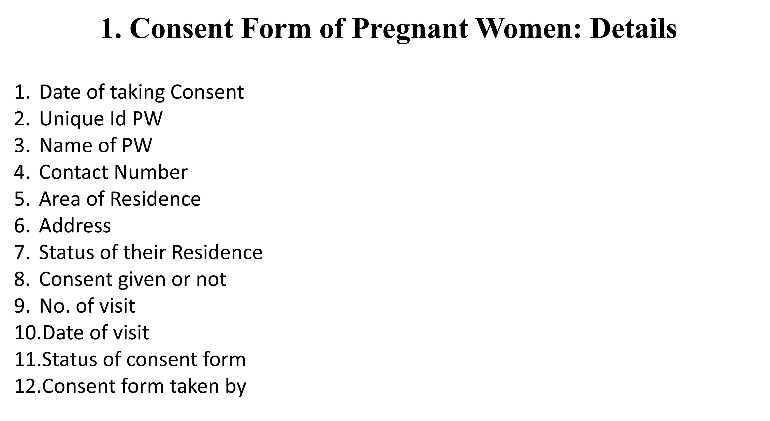


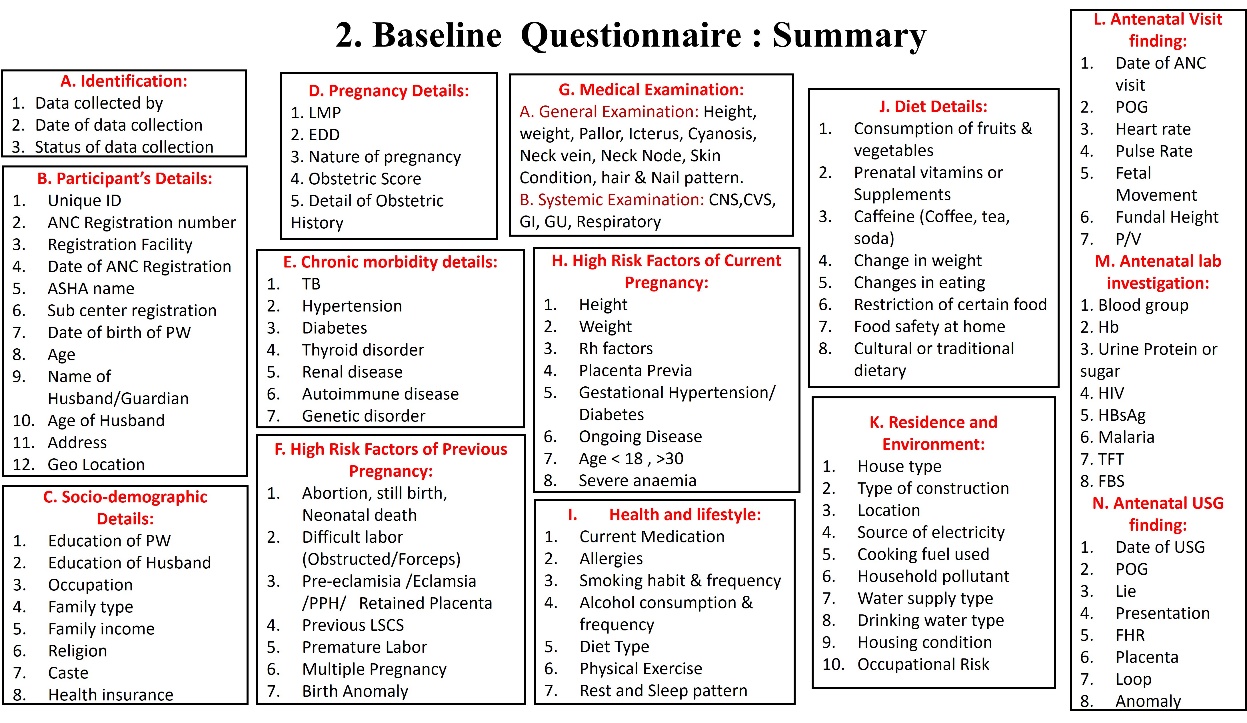


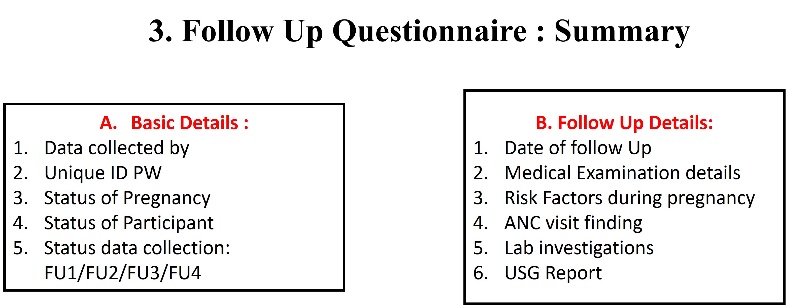


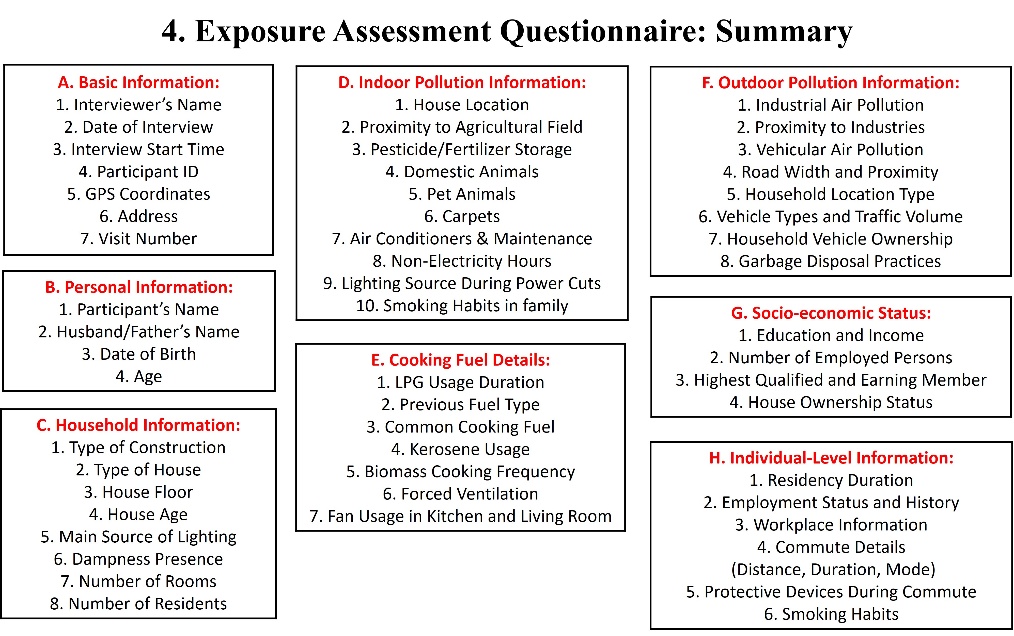


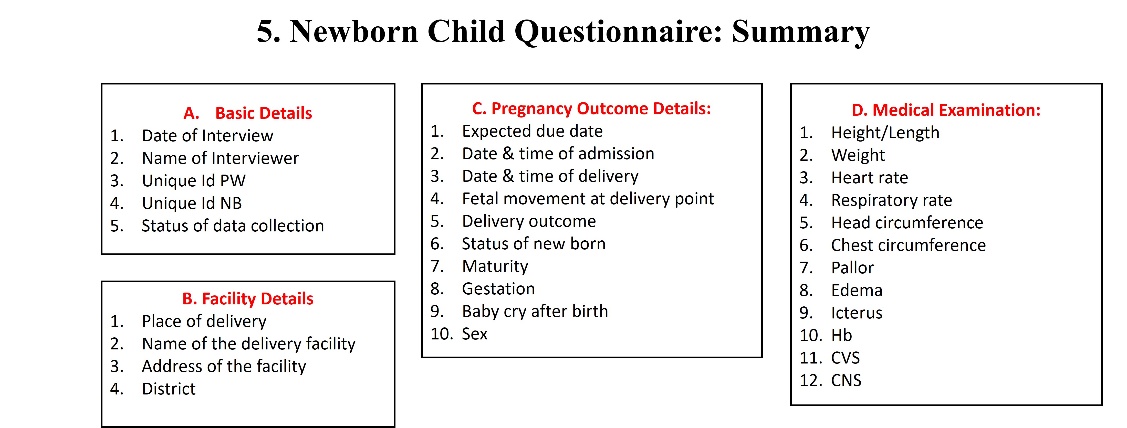

Supplement: Multimedia Appendix 1 [file resprot_v14i1e72683_app1.docx]
